# Baicalin promotes hippocampal neurogenesis via SGK1- and FKBP5-mediated glucocorticoid receptor phosphorylation in a neuroendocrine mouse model of anxiety/depression

**DOI:** 10.1038/srep30951

**Published:** 2016-08-09

**Authors:** Kuo Zhang, Xing Pan, Fang Wang, Jie Ma, Guangyue Su, Yingxu Dong, Jingyu Yang, Chunfu Wu

**Affiliations:** 1 Department of Pharmacology, Shenyang Pharmaceutical University, 110016, Shenyang, PR China; 2 Department of School of Functional Food And Wine, Shenyang Pharmaceutical University, 110016, Shenyang, PR China

## Abstract

Antidepressants increase hippocampal neurogenesis by activating the glucocorticoid receptor (GR), but excessive GR activation impairs hippocampal neurogenesis, suggesting that normal GR function is crucial for hippocampal neurogenesis. Baicalin was reported to regulate the expression of GR and facilitate hippocampal neurogenesis, but the underlying molecular mechanisms are still unknown. In this study, we used the chronic corticosterone (CORT)-induced mouse model of anxiety/depression to assess antidepressant-like effects of baicalin and illuminate possible molecular mechanisms by which baicalin affects GR-mediated hippocampal neurogenesis. We found that oral administration of baicalin (40, 80 or 160 mg/kg) for 4 weeks alleviated several chronic CORT-induced anxiety/depression-like behaviors. Baicalin also increased Ki-67- and DCX-positive cells to restore chronic CORT-induced suppression of hippocampal neurogenesis. Moreover, baicalin normalized the chronic CORT-induced decrease in GR protein levels, the increase in GR nuclear translocation and the increase in GR phosphorylation at Ser203 and Ser211. Finally, chronic CORT exposure increased the level of FK506-binding protein 51 (FKBP5) and of phosphorylated serum- and glucocorticoid-inducible kinase 1 (SGK1) at Ser422 and Thr256, whereas baicalin normalized these changes. Together, our findings suggest that baicalin improves anxiety/depression-like behaviors and promotes hippocampal neurogenesis. We propose that baicalin may normalize GR function through SGK1- and FKBP5-mediated GR phosphorylation.

Stress is the most powerful risk factor for the development of depression. Excessive and prolonged chronic stress results in hyperactivity of the hypothalamic-pituitary-adrenal (HPA) axis and hypersecretion of corticosteroids, which are the most consistent biological findings in major depression disorders^1^,^2^. Glucocorticoids are steroid hormones, which cross the blood-brain-barrier, and primarily bind to the high-affinity mineralocorticoid receptor (MR) and the low-affinity glucocorticoid receptor (GR)^1^. Because of its low affinity, GR is fully activated only when high concentrations of glucocorticoids are present, and GR is therefore considered to be particularly important in depression^3^. Recently, more and more evidence has consistently demonstrated that stress or high concentrations of glucocorticoids induce GR activation, which impairs hippocampal neurogenesis^4^,^5^,^6^,^7^. Importantly, hippocampal neurogenesis is an essential element of some of the behavioral effects of antidepressants, and this effect is activated by a GR-dependent mechanism^8^,^9^,^10^. However, the molecular signaling pathways involved in GR-mediated hippocampal neurogenesis are still unknown and await clarification.

Baicalin, one of predominant flavonoid compounds in Radix Scutellariae, has been shown to exhibit strong pharmacological effects, including anti-inflammation^11^, anti-cancer^12^, and anti-apoptosis^13^. Interestingly, it was recently reported that baicalin can facilitate the differentiation of neural stem/progenitor cells to neurons and stimulate hippocampal neurogenesis in adult rats^14^. In addition, baicalin was also reported to regulate the expression of GR^15^. These studies revealed the effects of baicalin on hippocampal neurogenesis and GR protein levels. GR is a ligand-activated transcription factor, which only executes its functions when it translocates from the cytoplasm to the nucleus^16^. Importantly, GR activation, GR nuclear translocation and GR-induced transcriptional changes are critically dependent on the phosphorylation status of specific serine (Ser) residues^9^. However, the effects of baicalin on GR nuclear translocation and phosphorylation have not yet been reported in detail.

To further illuminate the potential molecular mechanisms by which baicalin affects GR-mediated hippocampal neurogenesis, we firstly assessed the antidepressant-like effects of baicalin on behaviors and hippocampal neurogenesis in the mouse model of chronic CORT-induced anxiety/depression. We then characterized the effect of chronic CORT exposure and baicalin on GR level, GR nuclear translocation and GR phosphorylation in the hippocampus. Finally, to investigate the molecular mechanisms underlying the normalization of GR function by baicalin, we analyzed SGK1 and FKBP5, two proteins that are known to modulate GR function.

## Results

### Effects of baicalin on physical and behavioral changes in the CORT model of anxiety/depression

**Figure 1:**
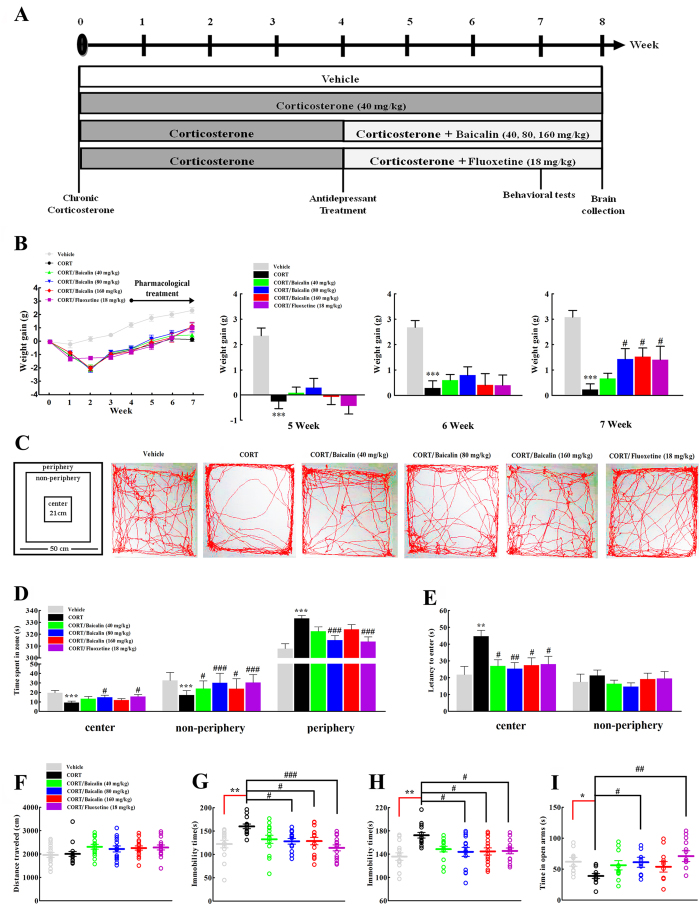
Effects of baicalin (40, 80 and 160 mg/kg) on different behaviors in the CORT model of anxiety/depression. A schematic representation of the experimental procedure is shown in (**A**). Graphs show the effect of baicalin on body weight (**B**), performance in the open field test (**C–E**), locomotor activity (**F**), immobility time in the tail suspension test (**G**), immobility time in the forced swimming test (**H**) and performance in the elevated plus maze (**I**). Baicalin significantly improves several anxiety and depression-like behaviors induced by chronic CORT treatment. Representative images of movement tracks in the open field test are shown in (**C**). Data are expressed as means ± S.E.M (*n* = 12–15 mice/group). *P < 0.05, **P < 0.01 and ***P < 0.001 *vs* vehicle. ^#^*P* < 0.05, ^##^*P* < 0.01 and ^###^*P* < 0.001 *vs* CORT model.

We used the mouse model of CORT-induced anxiety/depression to investigate the antidepressant-like effects of baicalin (40, 80, 160 mg/kg) in comparison with fluoxetine (18 mg/kg). Firstly, body weight gain was used to assess the physical changes induced by chronic CORT exposure. The results showed that chronic CORT exposure significantly slowed the gain in body weight compared to the vehicle-treated group (Fig. 1B, F(5, 84) = 14.561, *P* < 0.001). Interestingly, administration of baicalin (80 and 160 mg/kg) restored the body weight gain compared to CORT alone after 3 weeks of treatment (*P* = 0.028; *P* = 0.011, respectively). Moreover, in the open field test, baicalin increased the time spent in the center zone (Fig. 1D, F(5, 84) = 6.620, *P* = 0.023) and the non-periphery zone (Fig. 1D, F(5, 84) = 10.442, *P* < 0.05 or *P* < 0.001), and decreased the time spent in the periphery zone (Fig. 1D, F(5, 84) = 11.778, *P* < 0.001) and the latency to enter the center zone (Fig. 1E, F(5, 84) = 4.018, *P* = 0.021; *P* = 0.008; *P* = 0.029, respectively). These results demonstrated that baicalin can improve chronic CORT-induced anxiety-like behaviors. To rule out a false positive effect of psychostimulant drugs on locomotor activity, the distance traveled in the open field was measured and no differences were observed (Fig. 1F, F(5, 84) = 1.917, *P* = 0.999). In addition, chronic CORT exposure induced a decrease in the time spent in the open arms of the elevated plus maze (Fig. 1I, F(5, 66) = 3.972, *P* = 0.033), and this effect was reversed by baicalin (*P* = 0.047). Finally, the antidepressant-like effects of baicalin were evaluated by the tail suspension test and the forced swimming test, two behavioral despair models which are the most widely used for evaluating antidepressant activity^17^,^18^. Similar to fluoxetine, baicalin decreased the immobility time in the tail suspension test (Fig. 1G, F(5, 78) = 4.796, *P* = 0.026; *P* = 0.029, respectively) and the forced swimming test (Fig. 1H, F(5, 78) = 4.501, *P* = 0.012; *P* = 0.016, respectively), which indicates that baicalin can improve chronic CORT induced depression-like behaviors.

### Effects of baicalin on hippocampal proliferation and neurogenesis in the CORT model of anxiety/depression

**Figure 2:**
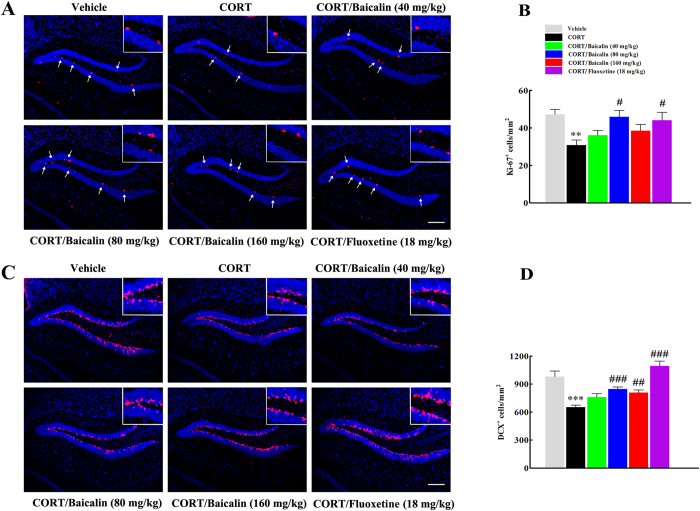
Effects of baicalin (40, 80 and 160 mg/kg) on hippocampal cell proliferation (Ki-67-positive cells) and neurogenesis (DCX-positive cells) in the CORT model of anxiety/depression. Representative images of Ki-67-positive cells (**A**) and DCX-positive cells (**C**) in the hippocampus are shown. Arrows in (**A**) indicate Ki-67-positive cells. Scale bars: 100 μm. Inserted images show Ki-67 and DCX immunostaining under high magnification (scale bar: 25 μm). Graphs show the density of positive cells for Ki-67 (**B**) or DCX (**D**) in the dentate gyrus. Baicalin significantly improves the chronic CORT-induced decrease in hippocampal neurogenesis. Data are expressed as means ± S.E.M (*n* = 6–8 mice/group). **P < 0.01 and ***P < 0.001 *vs* vehicle. ^#^*P* < 0.05, ^##^*P* < 0.01 and ^###^*P* < 0.001 *vs* CORT model.

Based on the critical role of hippocampal neurogenesis on stress responses and behavioral effects of antidepressants^10^,^19^, we investigated the potential mechanisms underlying the antidepressant effects of baicalin by evaluating hippocampal proliferation and neurogenesis in the CORT model. Firstly, we assessed the effect of baicalin on hippocampal proliferation by counting the density of Ki-67-positive cells. In accordance with previous research^5^,^6^, we found that chronic CORT exposure decreased the density of Ki-67-positive cells in the dentate gyrus of the hippocampus (Fig. 2B, F(5, 96) = 4.022, *P* = 0.006) and this reduction was reversed significantly by both baicalin (*P* = 0.014) and fluoxetine (*P* = 0.046). Next, we assessed the density of new-born neurons by counting DCX-positive cells in the dentate gyrus of the hippocampus. The results indicated that chronic CORT exposure decreased the density of DCX-positive cells (Fig. 2D, F(5, 102) = 15.242, *P* = 0.0008), whereas baicalin or fluoxetine reduced this decrease (*P* < 0.001; *P* = 0.002; *P* < 0.001, respectively).

### Effects of baicalin on glucocorticoid receptor levels and glucocorticoid receptor nuclear translocation in the CORT model of anxiety/depression

**Figure 3:**
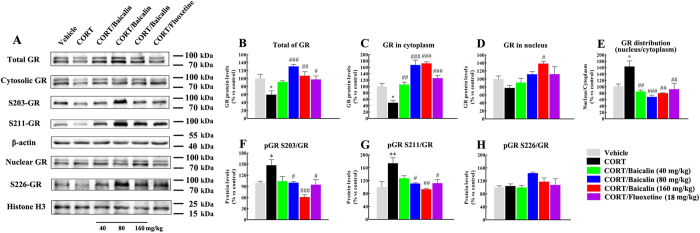
Effects of baicalin (40, 80 and 160 mg/kg) on the levels of GR and phosphorylated GR in the CORT model of anxiety/depression. Representative western blots of GR protein expression are shown (**A**). The graphs show quantification of total GR protein level (**B**), GR protein level in the cytoplasm (**C**), GR protein level in the nucleus (**D**), GR distribution (**E**), GR phosphorylation at S203 (**F**) and S211 (**G**) in the cytoplasm, and GR phosphorylation at S226 (**H**) in the nucleus. Baicalin significantly increases the total GR protein level and the cytoplasmic GR protein level, normalizes the GR distribution, and decreases the levels of pSer203/GR and pSer211/GR in the cytoplasm in the CORT model of anxiety/depression. All gels were run under the same experimental conditions and were cropped based on the molecular weight of the target protein (full-length blots are presented in Supplementary Figs S2–S9). The boundary between the gels is delineated by a black line. Data are expressed as means ± S.E.M (*n* = 6–8 mice/group). *P < 0.05 and **P < 0.01 *vs* vehicle. ^#^*P* < 0.05, ^##^*P* < 0.01 and ^###^*P* < 0.001 *vs* CORT model.

The glucocorticoid receptor plays an important role in depression and is involved in the effects of antidepressant drugs on neurogenesis^7^,^9^. To demonstrate whether the glucocorticoid receptor was changed by chronic CORT exposure, we firstly analyzed the expression of total GR protein in hippocampus. We found that chronic CORT significantly decreased the total GR protein level (Fig. 3B, F(5, 18) = 7.448, *P* = 0.033), and this change was normalized after baicalin treatment (*P* < 0.001; *P* < 0.01, respectively). Next, we analyzed the levels of GR protein in the cytosolic and nuclear compartments. As observed for total GR protein, chronic CORT exposure significantly decreased the GR protein level in the cytoplasm (Fig. 3C, F(5, 18) = 24.113, *P* = 0.013), and baicalin reversed this reduction (*P* = 0.005; *P* < 0.001; *P* < 0.001, respectively). On the other hand, chronic CORT exposure did not significantly change the GR protein level in the nucleus (Fig. 3D, F(5, 12) = 4.084, *P* = 0.644), but baicalin increased the GR protein level in the nucleus compared with the CORT model group (*P* = 0.013). It is noteworthy that chronic CORT exposure significantly increased the nuclear/cytoplasmic ratio of GR (Fig. 3E, F(5, 18) = 9.036, *P* = 0.011), whereas baicalin normalized this change in distribution (*P* < 0.01; *P* < 0.001; *P* < 0.01, respectively). To provide further insights into the mechanisms of GR nuclear translocation, we prepared cytoplasmic and nuclear fractions from hippocampal cells and investigated the phosphorylation status of the critical serine residues Ser203 and Ser211 of GR in the cytosolic compartment and Ser226 of GR in the nuclear compartment. Chronic CORT exposure significantly increased the levels of pSer203/GR (Fig. 3F, F(5, 18) = 7.119, *P* = 0.044) and pSer211/GR in the cytoplasm of hippocampal cells (Fig. 3G, F(5, 12) = 6.376, *P* = 0.007), and this increase was normalized by baicalin treatment (*P* < 0.05, *P* < 0.01 or *P* < 0.001). The level of pSer226/GR in the nucleus was not affected by chronic CORT treatment (Fig. 3H, F(5, 12) = 1.517, *P* = 0.999). Baicalin slightly increased the pSer226/GR level, but the change was not significant (*P* = 0.370).

### Effects of baicalin on FKBP5, SGK1 and SGK1 phosphorylation in the CORT model of anxiety/depression

**Figure 4:**
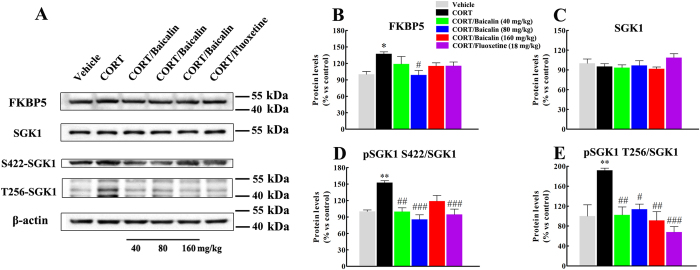
Effects of baicalin (40, 80 and 160 mg/kg) on the levels of FKBP5, SGK1 and phosphorylated SGK1 in the CORT model of anxiety/depression. Representative western blots of protein expression are shown (**A**). The graphs show quantification of FKBP5 protein level (**B**), SGK1 protein level (**C**), SGK1 phosphorylation at S422 (**D**) and SGK1phosphorylation at T256 (**E**). Baicalin significantly decreases the level of FKBP5, pSer422/SGK1 and pThr256/SGK1 in the CORT model of anxiety/depression. All gels were run under the same experimental conditions and were cropped based on the molecular weight of the target protein (full-length blots are presented in Supplementary Figs S10–S13). The boundary between the gels is delineated by a black line. Data are expressed as means ± S.E.M (*n* = 6–8 mice/group). *P < 0.05 and **P < 0.01 *vs* vehicle. ^#^*P* < 0.05, ^##^*P* < 0.01 and ^###^*P* < 0.001 *vs* CORT model.

In light of our finding that chronic CORT exposure changed GR protein levels, GR nuclear translocation and GR phosphorylation, we next assessed whether CORT treatment affected FKBP5 and SGK1, since these proteins are known to play critical roles in modulating GR function^7^,^16^,^20^,^21^. As shown in Fig. 4, chronic CORT significantly increased the level of FKBP5 in hippocampus (Fig. 4B, F(5, 12) = 3.236, *P* = 0.049), and baicalin normalized this change (*P* = 0.042). In contrast, chronic CORT treatment did not affect the level of SGK1 in hippocampus (Fig. 4C, F(5, 18) = 1.240, *P* = 0.987), but it is noteworthy that chronic CORT exposure significantly increased the levels of pSer422/SGK1 (Fig. 4D, F(5, 18) = 10.307, *P* = 0.001) and pThr256/SGK1 (Fig. 4E, F(5, 18) = 8.004, *P* = 0.005). Finally, baicalin normalized this increase (*P* < 0.05, *P* < 0.01 or *P* < 0.001).

## Discussion

In the present study, we used an optimized mouse model of anxiety/depression based on the elevation of glucocorticoids to investigate the antidepressant-like effects of baicalin. This model is more reliable, replicable and easily operable compared with stress models, and many studies have demonstrated that chronic exposure to corticosterone induces multiple anxiety/depression-like behaviors^5^,^6^. In line with previous studies^15^,^22^, our results showed that chronic CORT exposure induced several anxiety/depression-like physical and behavioral changes. These changes included slower body weight gain, decreased time spent in the center and non-periphery zone in the open-field test, decreased time spent in the open arms of the elevated plus maze, and in particular increased immobility time in the tail suspension test and the forced swimming test. Furthermore, we showed that baicalin treatment normalized these chronic CORT-induced anxiety/depression-related physical and behavioral changes. Our results revealed that high concentrations of glucocorticoids are connected with the pathophysiology of depression and indicate that baicalin possibly plays an important role in alleviating glucocorticoid-related depression.

Neurogenesis is related to some of the behavioral effects of antidepressants^8^. Some evidence suggests that neurogenesis occurs in several areas of the brain, including the hypothalamus, amygdala and cortex, etc, but general research has shown that new neurons are mainly found in the dentate gyrus of the hippocampus^23^. Moreover, the hippocampal formation is also critical for modulation of the hypothalamic-pituitary-adrenal axis and regulation of the stress response and emotion^1^,^2^. At present, definite mechanisms of hippocampal neurogenesis in depression are still vague, but hippocampal neurogenesis is considered as an essential element of some of the behavioral effects of antidepressants, and this has been verified by ablating hippocampal neurogenesis^5^,^10^. Based on these reasons, we investigated the effects of baicalin on hippocampal neurogenesis in the CORT model. A previous study showed that baicalin promoted hippocampal neurogenesis in a rat model of cerebral ischemic injury^14^ but not in a model of depression. Our findings showed that baicalin treatment reversed the CORT-induced decrease in cell proliferation and neurogenesis. The above results demonstrate that baicalin promotes hippocampal neurogenesis; however, the underlying molecular mechanisms are still unknown. Based on the critical role of GR in the HPA axis and in mediating the effects of glucocorticoids on the brain, it is not surprising that GR may be considered as a potential target for antidepressant drugs^24^. For example, the effect of antidepressants on neurogenesis requires normal glucocorticoid rhythms^25^, but excessive GR activation by high concentrations of glucocorticoids impairs hippocampal neurogenesis^1^,^8^, suggesting that normalization of GR function is the key to antidepressant action and hippocampal neurogenesis. In this context, we firstly assessed the effects of baicalin on total GR protein in hippocampus. Our results showed that total GR protein levels were decreased by chronic CORT exposure, suggesting that prolonged exposure to a GR agonist resulted in the down-regulation of GR expression. These results were also in accordance with studies *in vitro*, in which CORT decreases GR protein levels in whole cells^7^. In contrast, baicalin reversed this down-regulation of GR expression. As we know, GR is kept in the inactive state in the cytoplasm, and when it binds to glucocorticoids, it translocates from the cytoplasm to the nucleus to execute its functions^1^,^16^. However, the effects of baicalin on GR nuclear translocation have not been reported in detail. Therefore, to further study the functional mechanisms of GR, we investigated the effects of baicalin on GR nuclear translocation by analyzing the GR protein levels in the cytoplasm and nucleus. Chronic CORT exposure decreased the GR protein levels in the cytoplasm but not in the nucleus. It is noteworthy that chronic CORT exposure also changed the nuclear/cytoplasmic balance of GR distribution in hippocampus. These findings suggested that chronic CORT exposure decreased the cytosolic GR binding capacity and increased the level of active nuclear GR, which might weaken the feedback inhibition of the HPA axis and increase the negative effects of GR, thus impairing hippocampal neurogenesis. Our findings firstly showed that baicalin treatment restored the GR protein levels in the cytoplasm and nucleus, and in particular normalized the GR distribution, raising the possibility that baicalin had a positive effect on hippocampal neurogenesis by restoring normal GR function.

**Figure 5:**
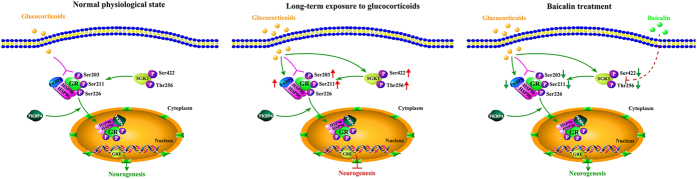
Proposed mechanisms of the action of baicalin and glucocorticoids in the SGK1- and FKBP5-mediated GR phosphorylation signaling pathway. In the normal physiological state, glucocorticoids activate GR and facilitate translocation of GR into the nucleus to regulate hippocampal neurogenesis. Prolonged exposure to high concentrations of glucocorticoids enhances SGK1 activity by increasing the phosphorylation of SGK1 at Ser422 and Thr256, which in turn induces the phosphorylation of GR at Ser203 and Ser211 to enhance nuclear translocation of GR. Meanwhile, high glucocorticoid levels also increase the expression of FKBP5, which weakens cytosolic GR binding capacity, impairs the ultra-short negative feedback loop on GR sensitivity and increases the negative effects of glucocorticoids. Baicalin may normalize FKBP5 protein levels and SGK1 phosphorylation at S422 and T256 to regulate GR phosphorylation, and then restore normal GR function to promote hippocampal neurogenesis.

Like many nuclear receptors, GR is a phosphoprotein, which maintains a basal level of phosphorylation and becomes hyperphosphorylated upon binding glucocorticoids^26^. Previous studies indicated that the phosphorylation of GR plays a critical role in mediating glucocorticoid signaling, including GR activation, nuclear translocation and gene transcription^9^,^16^. For this reason, we assessed the effects of baicalin on three important phosphorylation sites, Ser203 and Ser211 in cytoplasmic GR, and Ser226 in nuclear GR. Evidence from *in vitro* and *in vivo* studies has suggested that phosphorylation at sites Ser203 and Ser211 facilitate nuclear translocation and increase the transcriptional activities of the receptor, while conversely, phosphorylation at site Ser226 inhibits nuclear translocation and decreases GR transcriptional activities^7^,^16^,^26^. Our results showed that chronic CORT exposure increased GR phosphorylation at both Ser203 and Ser211, but had no observable effect at Ser226. It is noteworthy that baicalin significantly reduced phosphorylation at Ser203 and Ser211. These findings are in line with our results showing that baicalin normalized GR protein levels in the cytoplasm and nucleus, and suggest that baicalin might restore the balance of GR distribution by modulating GR phosphorylation. Finally, we analyzed the potential role of baicalin in two glucocorticoid- and antidepressant-responsive genes, the serum- and glucocorticoid-inducible kinase 1 (SGK1) and the FK506-binding protein 51 (FKBP5), which were recently demonstrated to play a vital part in mediating antidepressant action and hippocampal neurogenesis^21^,^27^. SGK1 belongs to a subfamily of serine/threonine kinases and regulates stress responses and neuronal function. Previous studies have shown that glucocorticoids increased SGK1 expression in human neural stem cells, whereas an inhibitor of SGK1 counteracted the glucocorticoid-induced reduction in neurogenesis, suggesting that SGK1 is a key link for the effects of glucocorticoids on neurogenesis and GR function^7^. *In vivo*, the expression of SGK1 in hippocampus under prolonged corticosterone exposure was not consistent in all studies^15^,^28^,^29^,^30^. This might be due to the wide variety of factors that control SGK1 transcription and the rapid degradation of SGK1^20^. In our study, no significant difference was found in the levels of hippocampal SGK1 protein in the different treatment groups, but chronic CORT exposure increased SGK1 phosphorylation at Ser422 and Thr256, and baicalin normalized these changes. It is noteworthy that SGK1 activity is controlled by phosphorylation, and maximal activation requires phosphorylation at both Ser422 in the hydrophobic motif and Thr256 in the T-loop^20^,^27^. The above results indicated that CORT increased the activity of SGK1 by increasing the phosphorylation at Ser422 and Thr256, which induced the phosphorylation of GR at Ser203 and Ser211 to enhance GR nuclear translocation^7^,^27^. FKBP5, a co-chaperone of Hsp90, has been reported to play a critical role in the regulation of GR sensitivity and GR phosphorylation^31^. In our study, chronic CORT exposure significantly increased the expression of FKBP5 in hippocampus, whereas baicalin reversed this change. This is in line with previous studies showing that chronic stress increased expression of FKBP5, which decreased cytosolic GR binding capacity and impaired feedback inhibition of the HPA axis^16^,^21^. Taken together, these findings revealed the potential molecular mechanisms by which baicalin is involved in SGK1- and FKBP5-mediated GR phosphorylation (Fig. 5). However, the precise target of baicalin requires further investigation in future studies.

In conclusion, we demonstrated that chronic corticosterone exposure induced several anxiety/depression-like behaviors in mice, whereas baicalin markedly alleviated these abnormal behaviors and promoted hippocampal neurogenesis. The effect of baicalin can probably be attributed to its ability to restore glucocorticoid receptor protein levels and normalize the nuclear translocation of the glucocorticoid receptor. The underlying molecular mechanisms probably involve SGK1- and FKBP5-mediated phosphorylation of the glucocorticoid receptor. Moreover, our results underline the crucial role of the glucocorticoid receptor in the pathophysiology of depression. Our findings open new perspectives on our understanding of the molecular mechanisms by which baicalin affects GR-mediated hippocampal neurogenesis, and on the development of unique approaches to antidepressant therapy.

## Methods

### Animals

Adult male C57BL/6J mice, aged between 6 and 7 weeks and weighing 18–22 g, were supplied by the Experimental Animal Centre of Shenyang Pharmaceutical University (Wenhua Road, Shenyang, PR China). Animals were housed in groups of six per cage under standardized environmental conditions (22 ± 2 °C, 12 h light/dark cycle with light on at 8:00 a.m.) with free access to food and water. All experiments were performed in accordance with relevant guidelines and regulations approved by the Experimental Animal Research Committee of Shenyang Pharmaceutical University. All efforts were made to minimize suffering and to reduce the number of animals used.

### Corticosterone model and drug treatment

The procedure for establishing the mouse model of CORT-induced anxiety/depression was performed as previously described^5^,^22^ with some modifications. Briefly, mice were subcutaneously injected daily for 8 weeks with corticosterone (40 mg/kg, TCI Development Co., Ltd, Japan) dissolved in sesame oil. Pharmacological treatment started in the 4th week after the beginning of the CORT protocol. Baicalin (40, 80, 160 mg/kg, Nanjing Zelang Medical Technology Co., Ltd, China, purity >99%) and fluoxetine (FLU, 18 mg/kg, Lilly S.A.) were dissolved in distilled water and given daily by gastric gavages 30 min prior to the corticosterone injection until the end of the experiment. The selected dose of baicalin in the present study was based on our previous studies (see Supplementary Fig. S1). The dose of fluoxetine was based on a previous report^5^. Behavioral tests were performed between the 7th and 8th weeks. At the end of the experiment, half of the mice (*n* = 8/group) were anesthetized with sodium pentobarbital (50 mg/kg, intraperitoneally) and transcardially perfused before the brains were collected for immunohistochemistry. The other half of the mice (*n* = 8/group) were sacrificed and the brain regions were rapidly dissected on dry ice and stored at −80 °C for further analysis. All procedures are shown in Fig. 1A.

### Behavioral tests

Mice were tested with four different behavioral tests of anxiety and depression, and a video tracking system (Ethovision, Noldus Systems, Wageningen, The Netherlands) was used to record animal behavior and analyse the data.

#### Open field test

The open field test was performed as described previously with minor modifications^32^. The open field was square in shape (50 cm × 50 cm) and contained three defined zones, the periphery zone, the non-periphery zone and the centre zone (21 cm × 21 cm). Briefly, mice were placed in the corner of the open field, within the periphery zone. Mice were allowed to explore the open field for 6 min. The movements of the mice were tracked by a video camera and the videos were analyzed with the video tracking system (Ethovision, Noldus Systems, Wageningen, The Netherlands). In accordance with previous studies^32^, the tracking system automatically recognized the mice and recorded the latency time to enter the two other areas (the non-periphery zone and center zone) and the time spent in the three areas under dim lighting (Fig. 1C). After each test, the open field was cleaned with ethanol to remove olfactory cues.

#### Elevated plus maze

The elevated plus maze was performed as previously described^33^. Mice were placed in the center area facing one of the closed arms, and then allowed to explore the maze for 5 min. The movements of the mice were tracked by a video camera and the videos were analyzed with the video tracking system (Ethovision, Noldus Systems, Wageningen, The Netherlands). In accordance with previous studies^33^, the tracking system automatically recognized the mice and recorded the time spent in the different arms. After each test, the maze was cleaned with ethanol to remove olfactory cues.

#### Tail suspension test

The tail suspension test was used as previously described^18^. Mice were individually suspended by the tail with adhesive tape on the edge of a metal rod 50 cm above a table. Mice were considered immobile only when they were completely motionless. Each mouse was suspended for a total of 6 min, and the immobility time was measured during the final 4 min of the test. The movements of the mice were tracked by a video camera and the videos were analyzed with the video tracking system (Ethovision, Noldus Systems, Wageningen, The Netherlands). The tracking system automatically recognized the mice and recorded the immobility time during the final 4 min of the test. Testing was carried out in a darkened room, avoiding background noise.

#### Forced swimming test

The forced swimming test was performed as previously described^17^. Mice were individually placed in eight plastic cylindrical tanks (height 40 cm, diameter 12 cm) filled to a depth of 10 cm with water (24 ± 1 °C) for 6 min. Each mouse was judged as immobile when it ceased struggling and remained floating motionless in the water, making only those movements necessary to keep its head above water. The movements of the mice were tracked by a video camera and the videos were analyzed with the video tracking system (Ethovision, Noldus Systems, Wageningen, The Netherlands). The tracking system automatically recognized the mice and recorded the immobility time during the final 4 min of the test. The water was changed after each trial to avoid any influence of excreted substances.

### Immunohistochemistry

Anesthetized mice (sodium pentobarbital, 50 mg/kg, i.p.) were transcardially perfused with saline solution followed by 4% paraformaldehyde in phosphate buffer (0.1 M, pH 7.4). Brains were removed, post-fixed overnight in 4% paraformaldehyde at 4 °C, and cryoprotected in 20% and 30% sucrose solution respectively at 4 °C. Then, the tissue was sectioned on a freezing microtome (AS-620, Shandon, Astmoor, UK) at a thickness of 25 μm (bregma −1.80 to −1.90 mm) for immunohistochemical procedures. The sections were adapted to room temperature, rehydrated and blocked with 5% NGS for 1 h at 37 °C, and then incubated overnight at 4 °C with primary antibody (rabbit anti-Ki-67 antibody, 1:1000, Abcam; rabbit anti-DCX antibody, 1:1000, Abcam), and subsequently incubated for 1 h with secondary antibody (Cy3-conjugated goat anti-rabbit IgG, Beyotime Institute of Biotechnology). Finally, the sections were washed in 0.1 M PBS and cover-slipped with anti-fade mounting medium (Beyotime Institute of Biotechnology). Images of Ki-67- and DCX-positive cells in the dentate gyrus of the hippocampus (bregma −1.80 to −1.90 mm) were digitized using an Olympus BX40 microscope (Olympus, Tokyo, Japan) fitted with a x10 objective lens. Then, Ki-67 and DCX-positive cells were automatically quantified using Image J software followed the threshold which could isolate positive cells from background. The density of Ki-67 and DCX-positive cell in the dentate gyrus was calculated in three brain sections per animal. Sections were viewed with a x10 objective lens.

### Western blot analysis

At the end of the experiment, mice were sacrificed, the brains were rapidly removed, and the hippocampus was dissected on an ice-chilled glass plate. Tissues were manually homogenized in RIPA buffer (Cell Signaling Technology, Danvers, MA) in the presence of protease inhibitor (PMSF) and phosphatase inhibitor (Na-ortho-vanadate, NaF). Nuclear and cytosolic extracts were obtained using a nuclear and cytoplasmic protein extraction kit (Beyotime Institute of Biotechnology) according to the manufacturer’s instructions. Protein concentrations were quantified by the bicinchoninic acid method, and equal amounts of protein (20–25 mg) were separated by 8–10% SDS-polyacrylamide gels and then electrophoretically transferred onto PVDF membranes (Millipore). Non-specific binding sites were blocked in 5% non-fat milk for 1 h and then the membranes were incubated with primary antibodies at 4 °C overnight, including mouse anti-GR (1:1000, Abcam), rabbit anti-phospho-GR (S203, 1:1000, Abcam), rabbit anti-phospho-GR (S211, 1:1000, Cell Signaling), rabbit anti-phospho-GR (S226, 1:500, Abcam), rabbit anti-SGK1 (1:1000, Abcam), rabbit anti-phosphor-SGK1 (S422, 1:1000, Abcam), rabbit anti-phosphor-SGK1 (T256, 1:1000, Santa Cruz), rabbit anti-FKBP5 (1:1000, Abcam), mouse anti-β-actin (1:1000, Santa Cruz) and mouse anti-histone H3 (1:1000, Abcam). Following incubation with the appropriate secondary antibody, the membranes were visualized by chemiluminescence using ECL detection reagents (GE Healthcare Europe GmbH) and exposed to X-ray films. Results were normalized to the internal control β-actin or histone H3 and the optical density was quantified by Image-J.

### Statistical analyses

Results from data analyses are expressed as the mean ± S.E.M. The data were compared by one-way ANOVA performed with Tukey’s HSD post hoc comparison. Statistical analysis was carried out using SPSS 19.0 software for Windows (SPSS Inc, Chicago, IL, USA). *P* < 0.05 was set as statistically significant.

## Additional Information

**How to cite this article**: Zhang, K. *et al*. Baicalin promotes hippocampal neurogenesis via SGK1- and FKBP5-mediated glucocorticoid receptor phosphorylation in a neuroendocrine mouse model of anxiety/depression. *Sci. Rep.* **6**, 30951; doi: 10.1038/srep30951 (2016).

## Supplementary Material

Supplementary Information
