# Supplementary material for: Baicalin promotes hippocampal neurogenesis via SGK1- and FKBP5-mediated glucocorticoid receptor phosphorylation in a neuroendocrine mouse model of anxiety/depression
Source: Sci Rep. 2016 Aug 9;6:30951. doi: 10.1038/srep30951 (PMC4977505; doi:10.1038/srep30951)
Supplement: Supplementary Information [file srep30951-s1.pdf]

Baicalin promotes hippocampal neurogenesis via SGK1- and FKBP5-mediated glucocorticoid receptor phosphorylation in a neuroendocrine mouse model of anxiety/depression

Kuo Zhang[a], Xing Pan[a], Fang Wang[a], Jie Ma[a], Guangyue Su[b], Yingxu Dong[a], Jingyu Yang[a,*], Chunfu Wu[a, **]

[a] *Department of Pharmacology, Shenyang Pharmaceutical University, 110016, Shenyang, PR China*

[b] *Department of School of Functional Food And Wine, Shenyang Pharmaceutical University, 110016, Shenyang, PR China*

* Corresponding author at: Department of Pharmacology, Shenyang Pharmaceutical University, Box 31, 103 Wenhua Road, 110016, Shenyang, PR China. Tel. & Fax: +86 24 23986339. E-mail address: yangjingyu2006@gmail.com

** Corresponding author at: Department of Pharmacology, Shenyang Pharmaceutical University, Box 31, 103 Wenhua Road, 110016, Shenyang, PR China. Tel. & Fax: +86 24 23986339. E-mail address: wucf@syphu.edu.cn

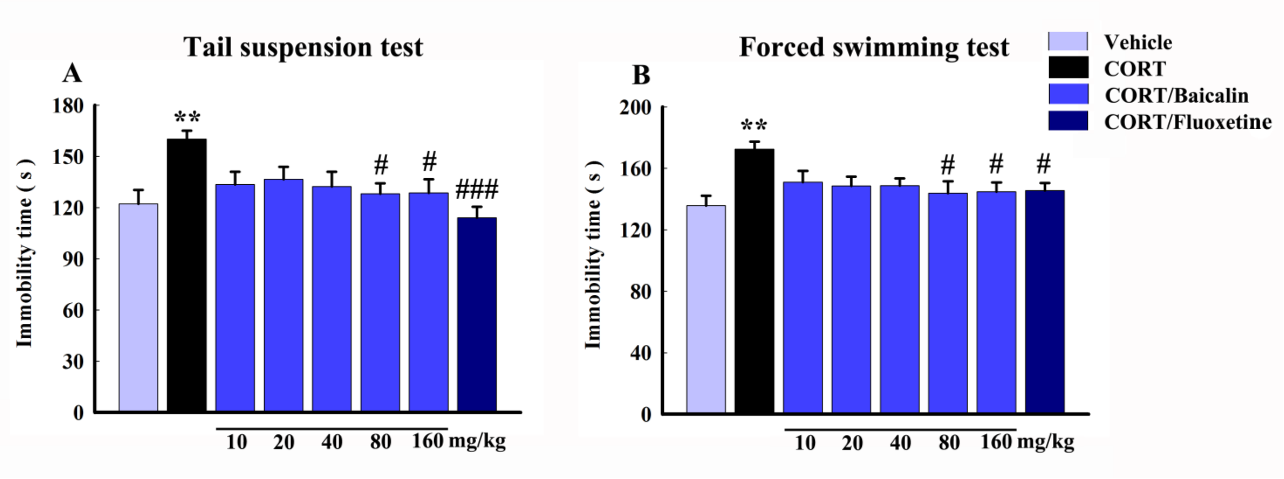

Supplementary Fig. S1 Effects of baicalin (40, 80 and 160 mg/kg) in the CORT model of anxiety/depression on immobility time in the tail suspension test (A) and the forced swimming test (B). Data are expressed as means ± S.E.M (n = 12-15 mice/group). $^{**}P < 0.01$ *vs* vehicle. $^{\#}P < 0.05$ and $^{\#\#\#}P < 0.001$ *vs* CORT model.

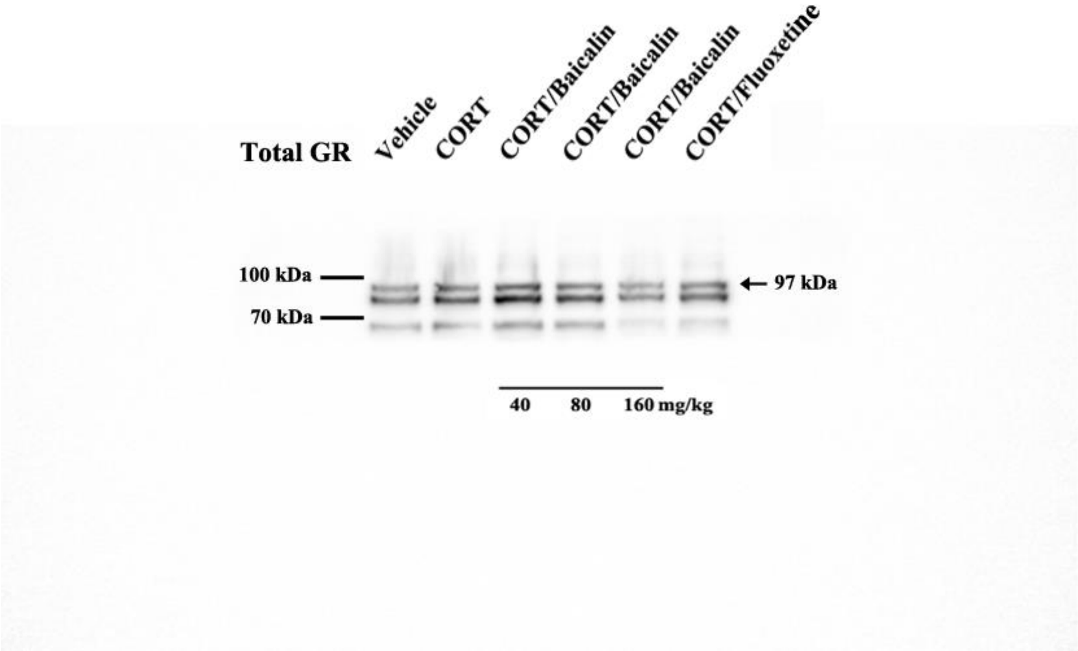

Supplementary Fig. S2 Representative blots of total GR in hippocampus in the CORT model of anxiety/depression

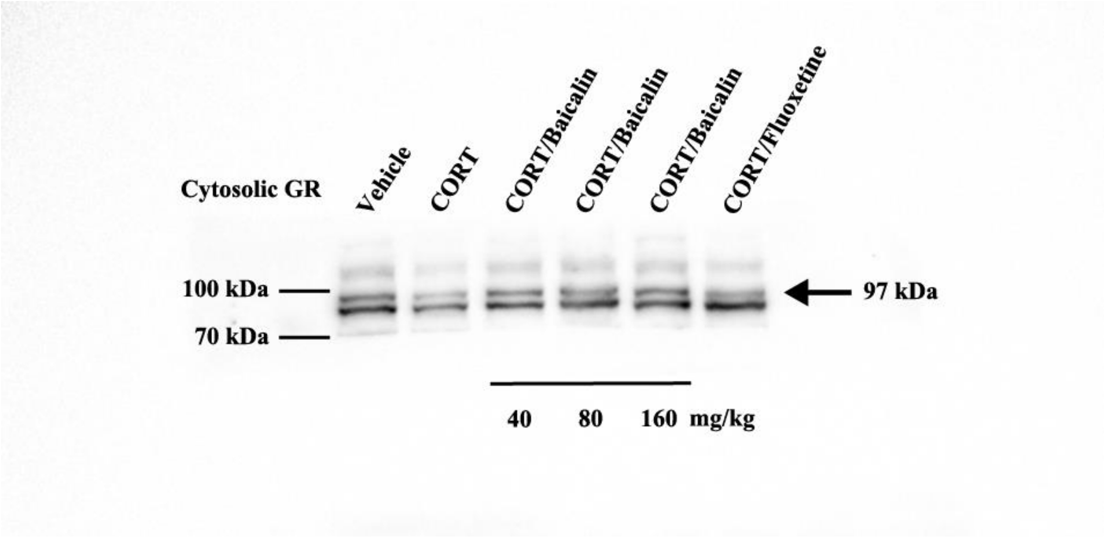

Supplementary Fig. S3 Representative blots of cytosolic GR in hippocampus in the CORT model of anxiety/depression

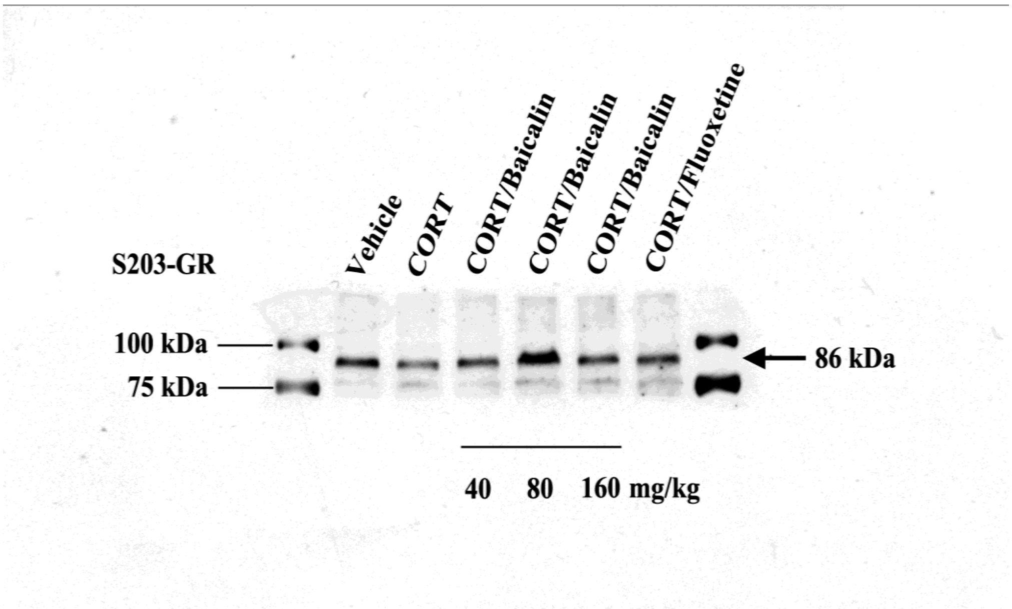

Supplementary Fig. S4 Representative blots of S203-GR in hippocampus in the CORT model of anxiety/depression

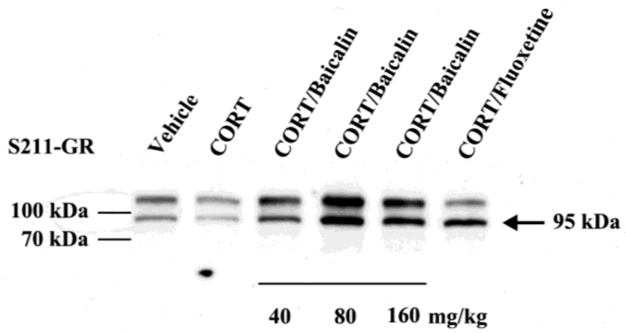

Supplementary Fig. S5 Representative blots of S211-GR in hippocampus in the CORT model of anxiety/depression

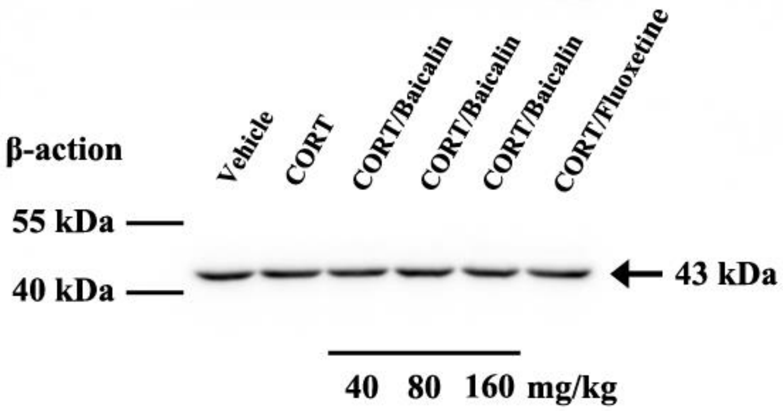

Supplementary Fig. S6 Representative blots of β-action in hippocampus in the CORT model of anxiety/depression

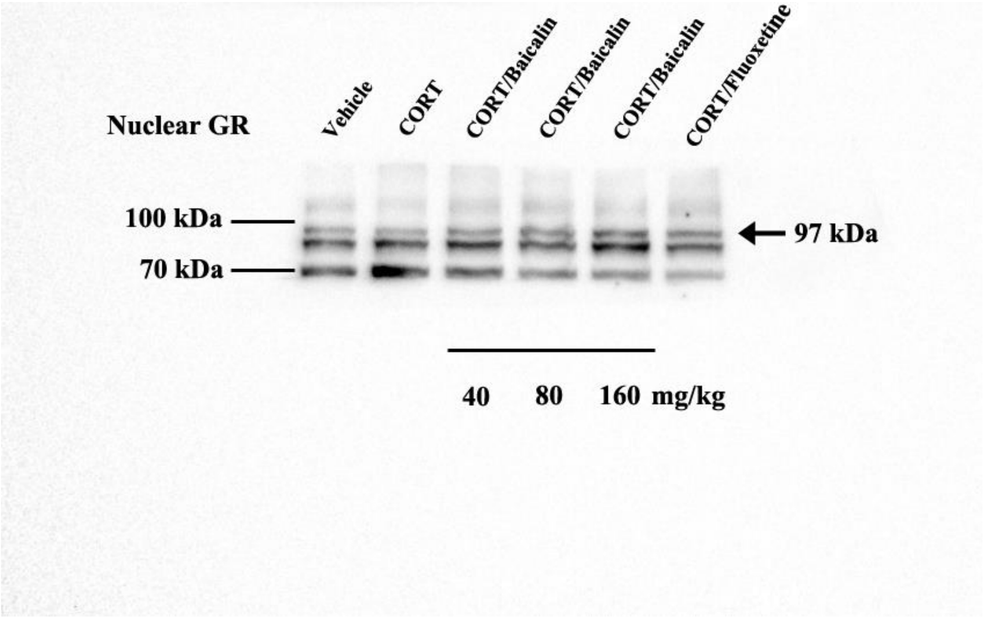

Supplementary Fig. S7 Representative blots of Nuclear GR in hippocampus in the CORT model of anxiety/depression

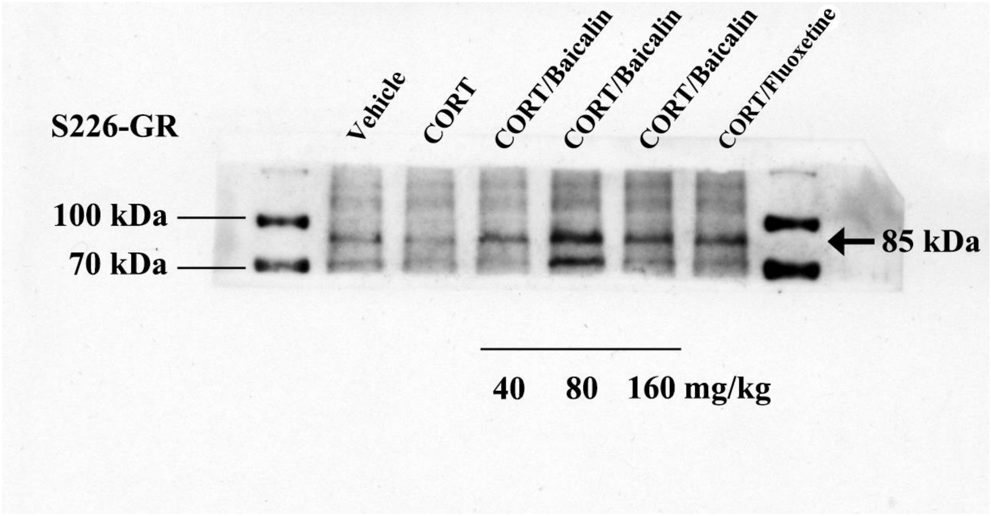

Supplementary Fig. S8 Representative blots of S226-GR in hippocampus in the CORT model of anxiety/depression

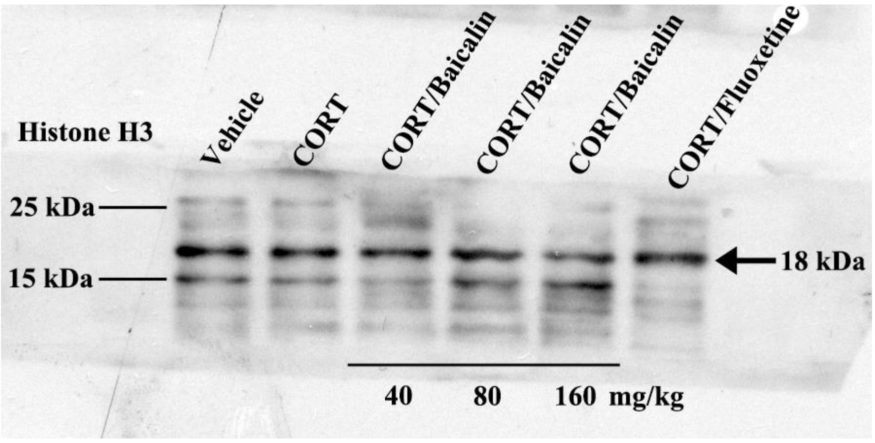

Supplementary Fig. S9 Representative blots of Histone H3 in hippocampus in the CORT model of anxiety/depression

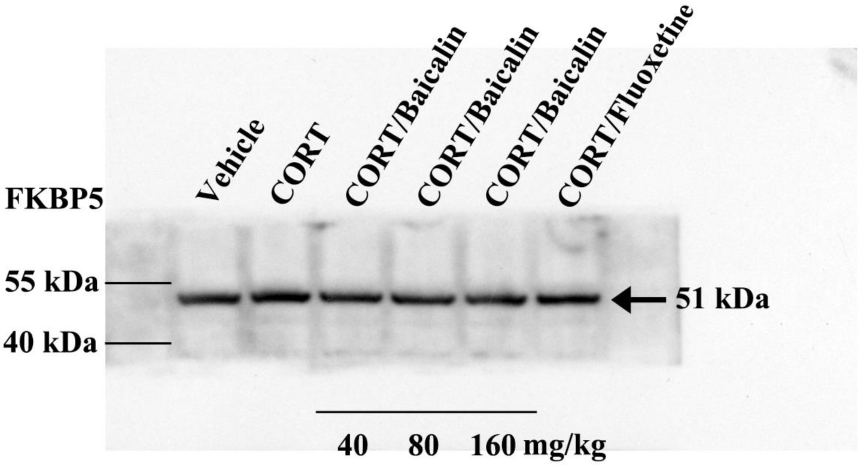

Supplementary Fig. S10 Representative blots of FKBP5 in hippocampus in the CORT model of anxiety/depression

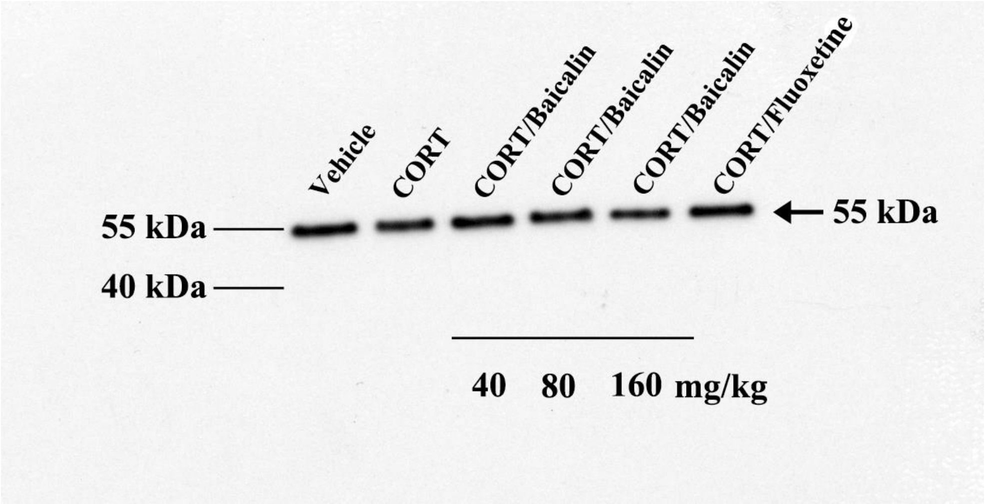

Supplementary Fig. S11 Representative blots of SGK1 in hippocampus in the CORT model of anxiety/depression

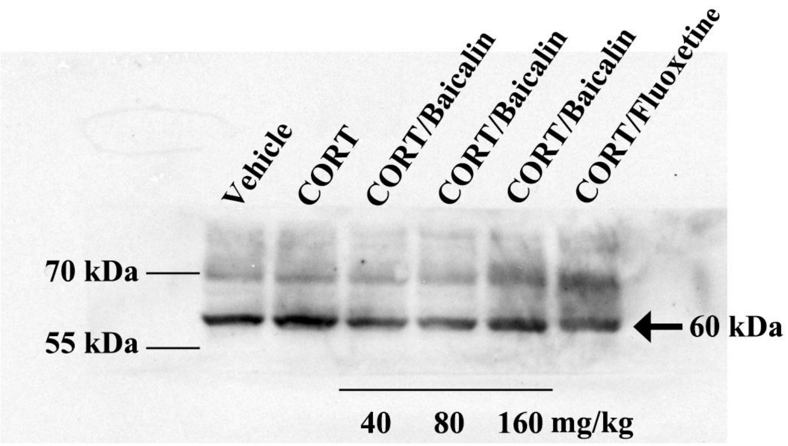

Supplementary Fig. S12 Representative blots of S422-SGK1 in hippocampus in the CORT model of anxiety/depression

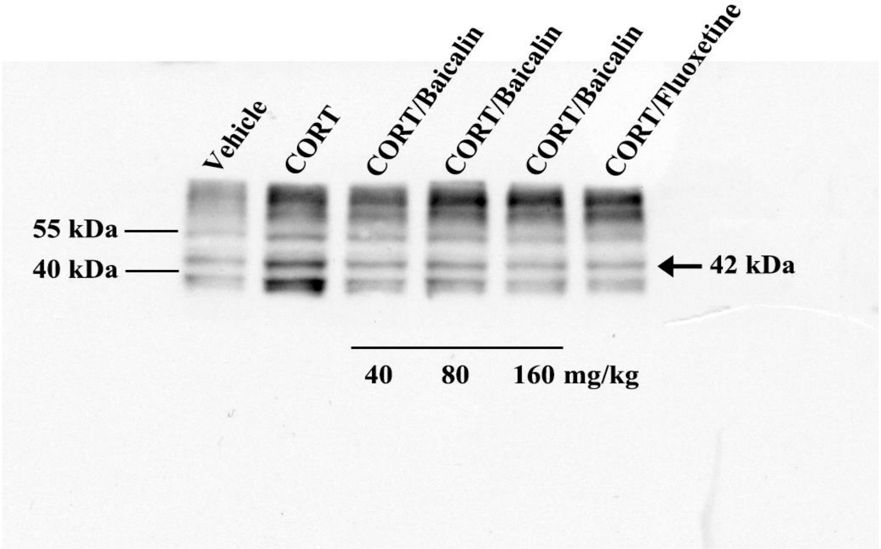

Supplementary Fig. S13 Representative blots of T256-SGK1 in hippocampus in the CORT model of anxiety/depression
